# Multi-Fingerprints Indoor Localization for Variable Spatial Environments: A Naive Bayesian Approach

**DOI:** 10.3390/s24185940

**Published:** 2024-09-13

**Authors:** Chengjie Hou, Zhizhong Zhang

**Affiliations:** 1 School of Communication and Information Engineering, Chongqing University of Posts and Telecommunications, Chongqing 400000, China; d190101003@stu.cqupt.edu.cn; 2 School of Electronic and Information Engineering, Nanjing University of Information Science and Technology, Nanjing 210000, China

**Keywords:** indoor localization, multi-fingerprints, naive Bayesian, Wi-Fi fingerprints

## Abstract

Fingerprint-based indoor localization has been a hot research topic. However, the current fingerprint-based indoor localization approaches still rely on a single fingerprint database, where the average level of data at reference points is used as the fingerprint representation. In variable environmental conditions, the variations in signals caused by changes in the environmental states introduce significant deviations between the average level and the actual fingerprint characteristics. This deviation leads to a mismatch between the constructed fingerprint database and the real-world conditions, thereby affecting the effectiveness of fingerprint matching. Meanwhile, the sharp noise interference caused by uncertainties such as personnel movement has a significant interference on the creation of the fingerprint database and fingerprint matching in online stage. Examination of the sampling data after denoising with Robust Principal Component Analysis (RPCA) revealed distinct multi-fingerprint characteristics with clear boundaries at certain access points. Based on these observations, the concept of constructing a fingerprint database using multiple fingerprints is introduced and its feasibility is explored. Additionally, a multi-fingerprint solution based on naive Bayes classification is proposed to accurately represent fingerprint characteristics under different environmental conditions. This method is based on the online stage fingerprints. The corresponding state space is selected using the naive Bayes classifier, enabling the selection of an appropriate fingerprint database for matching. Through simulations and empirical evaluations, the proposed multi-fingerprints construction scheme consistently outperforms the traditional single-fingerprint database in terms of positioning accuracy across all tested localization algorithms.

## 1. Introduction

In recent years, with the rapid development of mobile internet and smart devices, the demand for Location-Based Services (LBS) [1] has been increasing. LBS provides personalized services to users, such as indoor navigation, location-based recommendations, and social networking, among others. Although Global Navigation Satellite Systems (GNSS) [2] can provide reliable and high-precision positioning services in outdoor environments, they are limited in providing indoor positioning services due to factors such as building obstructions, signal interference, and multipath effects in complex indoor environments. Due to the low cost and widespread deployment of Wi-Fi devices, Wi-Fi-based indoor positioning technology has been a hot topic of research [3,4,5,6].

In indoor localization, positioning methods can be broadly categorized into range-based and range-free methods. Range-based methods depend on measuring the distance between the user and reference points or base stations through techniques like time of arrival (TOA) [7], angle of arrival (AOA) [8], and time difference of arrival (TDOA) [9]. These range-based positioning methods generally offer relatively high accuracy but require additional hardware support and complex deployment, and they are susceptible to the effects of non-line-of-sight (NLOS) signal transmission. In contrast, range-free positioning methods based on fingerprint matching are less affected by NLOS signal transmission and typically do not require the deployment of additional hardware devices. Currently, there are various data sources that can be used in fingerprint-based indoor localization solutions, including channel state information (CSI) [10], geomagnetic signals [11], LED signals [12], and received signal strength indicator (RSSI) [13,14]. Among them, Wi-Fi RSSI is easily obtainable and does not require additional equipment, making it a popular choice.

Wi-Fi fingerprint-based indoor localization algorithms involve two phases: offline and online [15]. During the offline phase, RSSI signals from Wi-Fi access points (APs) are collected at various reference points (RPs) and processed to create a fingerprint database, which serves as the foundation for the fingerprint positioning system. In the online stage, a user’s online fingerprint data are compared with the offline database to identify similar reference points. The target location of the user is then estimated by calculating a weighted average of these positions of reference points. In the early stage of data collection and processing [16], we found that a single fingerprint cannot represent the variations in different environmental states accurately. This deviation from reality affects the effectiveness of fingerprint matching. This impact becomes more pronounced when there are significant changes in the propagation paths of Wi-Fi signals due to spatial environment variations, such as transitioning from penetration-only to diffraction or even line-of-sight propagation.

Fingerprint-based indoor localization has significantly advanced since its inception. The first system, RADAR [17], developed by Bahl et al., collects RSSI at multiple receivers and uses empirical triangulation for localization. Moustafa et al. introduced the Horus system, which enhances positioning accuracy with a probabilistic K-nearest neighbors (KNN) approach [18]. Building on this, weighted K-nearest neighbors (WKNN) considers the weights among RPs [19]. In addition, there are many machine learning-based indoor fingerprint positioning solutions, such as CiFi [20] proposed by Wang et al. and Confi [21] proposed by Chen et al. Although research on fingerprint-based positioning has matured significantly, the focus has long been primarily on single fingerprints, and there is still a lack of research related to multi-fingerprints. The MFMCF system [22] proposed by Yuan et al. introduces the concept of multi-fingerprints, but these multi-fingerprints essentially involve the fusion of different data sources rather than the features of the same data source under different conditions. Yu et al. propose a solution for time-varying environments [23], where fingerprint databases are constructed separately for different time periods of the day, but this approach can be considered as a pseudo multi-fingerprints method. While Yu et al. considered complex pedestrian environments by training models with data collected under different pedestrian densities [24], they did not take into account the impact of complex spatial environments, which is far more significant than the influence of pedestrians.

In data processing, the interference of sharp noise has always been the primary issue in constructing precise offline and online fingerprints. The most common methods for noise reduction include Gaussian filtering, median filtering, and Kalman filtering. Fang et al. were the first to utilize Principal Component Analysis (PCA) for processing fingerprint databases, aiming to achieve more accurate fingerprint matching [25]. However, PCA performs poorly in datasets with many outliers. Candés extended PCA by considering the impact of sparse noise and developed Robust Principal Component Analysis (RPCA) [26]. RPCA decomposes the original matrix into a low-rank clean matrix and a matrix composed of sparse noise. Previously, RPCA has been widely used in image analysis, achieving notable success in low-rank image processing. Zhang et al. explored applying RPCA to update a Wi-Fi-based fingerprint database [27], and Li et al. proposed TILoc [28] using the same method. However, directly applying RPCA to the entire fingerprint database may have drawbacks, as each entry in the Wi-Fi fingerprint database is independent, while RPCA processes it as a unified whole. Nevertheless, the outliers modeling proposed by RPCA closely aligns with the actual situation of the collected RSSI data. Therefore, the RPCA algorithm is an effective method for denoising RSSI sampling data.

Based on the above, this paper proposes a concept of multi-fingerprints specifically designed for complex spatial environments. It maps different spatial environmental states between APs and RPs to different fingerprints in order to construct a multi-fingerprints database. Then, in the online phase, a naive Bayes classifier is employed to identify the real-time fingerprint and associate it with the corresponding state space, thereby improving positioning accuracy. The main contributions of this paper can be briefly described as follows:

- This paper utilizes RPCA to filter sharp noise from RSSI sampled data. By observing the denoised data through RPCA, clear data boundaries were identified between certain data, making them easily distinguishable. Therefore, this paper proposes the concept of multi-fingerprints, suggesting that the fingerprint of the same reference point can be represented by multiple data.
- A concept of multi-fingerprints is introduced for different spatial environmental states. The variability of the spatial environment states makes it impractical to represent all the state characteristics between each reference point and the APs using a single fingerprint. Therefore, it is necessary to consider the changes in spatial environmental states in order to construct an appropriate multi-fingerprints database.
- The use of the naive Bayes method is proposed to discriminate the spatial state of the online fingerprint, enabling the selection of the corresponding fingerprint from the multi-fingerprints database for matching.
- Simulation and experimental verification demonstrate that the variation in spatial environmental states has a significant impact on the propagation of AP signals. Moreover, the proposed multi-fingerprints approach outperforms the use of a traditional single fingerprint database in terms of localization accuracy.

The remainder of this paper is organized as follows. Section 2 presents the overall framework of the model. Section 3 provides a detailed description of the proposed localization scheme. Section 4 presents the analysis of the simulation and experimental results. Finally, Section 5 concludes the paper.

## 2. System Framework

To make a clear description of the system framework, it is necessary to provide special notation for basic symbols. It is assumed that there are *N* reference points, *M* access points, and *S* state spaces in the sampling area, with the distance between adjacent reference points denoted as *d*. The position of the *i*-th RP can be represented as $l_{i}=\left(x_{i},y_{i}\right)$, while the vector of RSSI from *M* APs at *i*-th RP in *j*-th state space can be represented as follows:

(1) $$\phi_{ij}=\left(rss_{i1}^{j},rss_{i2}^{j},\cdots ,rss_{iM}^{j}\right),$$

where $rss_{iM}^{j}$ represents the RSSI value in the *j*-th state space, sampled from the *M*-th AP at the *i*-th RP, i∈{1,2,⋯,N} and j∈{1,2,⋯,S}. The dataset of all location information is denoted as $L=(l_{1},l_{2}\cdots ,l_{N})T$, and all RSSI information in the *j*-th state space obtained by all RPs is as follows:

(2) $$\Phi_{j}=\left(\begin{array}{c} \phi_{1j}\\ \phi_{2j}\\ \vdots \\ \phi_{Nj} \end{array}\right)=\left(\begin{array}{c} rss_{11}^{j},rss_{12}^{j},\cdots ,rss_{1M}^{j}\\ rss_{21}^{j},rss_{22}^{j},\cdots ,rss_{2M}^{j}\\ \vdots \\ rss_{N1}^{j},rss_{N2}^{j},\cdots ,rss_{NM}^{j} \end{array}\right).$$

**Figure 1:**
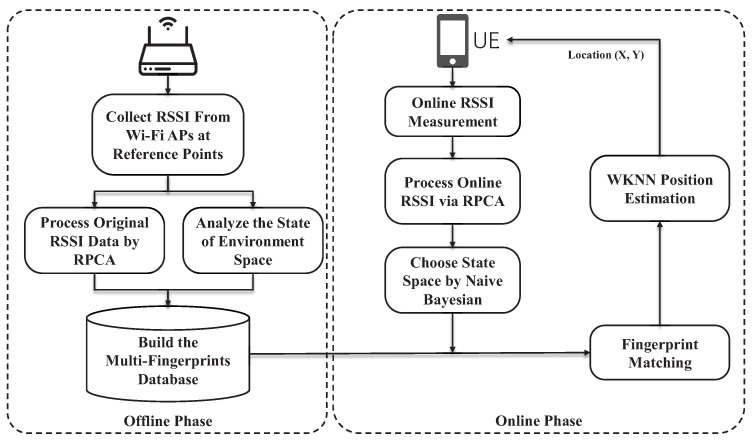
Multi-state spaces indoor localization system architecture based on multi-fingerprints.

The localization scheme proposed in this paper, which takes into account complex spatial states, is still based on a fingerprint localization system. As such, its main architecture includes both an offline phase and an online phase. The system framework is illustrated in Figure 1.

### 2.1. Offline Phase

The offline phase involves the construction of a multi-fingerprints database containing all possible spatial states of the environment. RSSI sampling data are collected from all APs at each reference point. RPCA is employed to process all the raw RSSI sample data, and the inexact augmented Lagrange multiplier (IALM) solver [29] is utilized to obtain the denoised, clean data.

**Figure 2:**
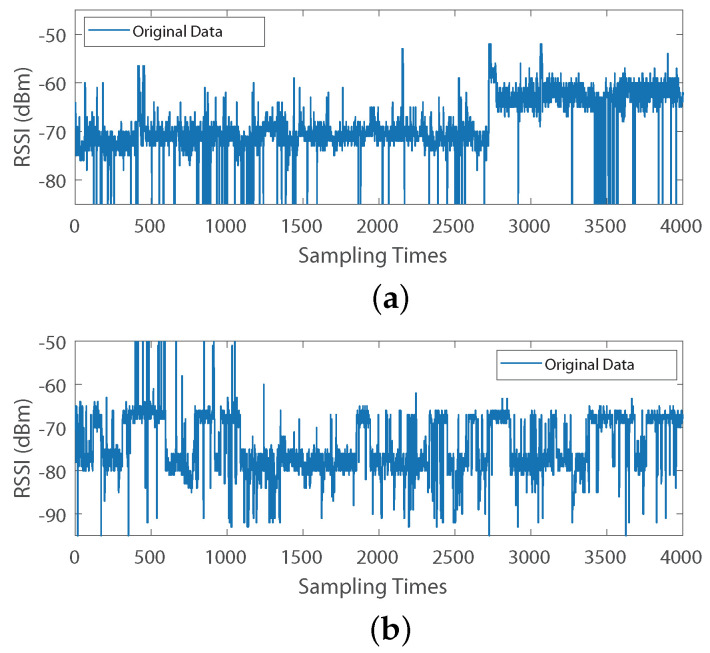
(**a**,**b**) The display of 4000 sample data of two APs.

As can be seen from Figure 2, using the overall data mean as the sole fingerprint to measure an AP’s performance reflects only the average case and may not accurately represent the AP’s behavior under different conditions. For example, in Figure 2a, the AP is clearly stable in two different states during the first and second halves, respectively, whereas in Figure 2b, the AP switches between two different states frequently. This is because the experiment room’s doors were in two different states, open and closed, which caused certain AP signals to transition from penetrating wall attenuation to potentially stronger transmission paths, such as direct line-of-sight transmission. Therefore, it is necessary to analyze the spatial environment states to construct a multi-fingerprints database that is different from the traditional fingerprint database [16]. It is also evident that a substantial amount of sparse noise exists in the raw sampled data. Therefore, it is essential to perform RPCA denoising on the sampled data.

### 2.2. Online Phase

In the online phase, the collected online data are also processed by RPCA denoising to construct online fingerprint. Then, naive Bayesian is used to classify the online fingerprint, which could find the corresponding state space. Online data are matched with the offline fingerprint data of the state space selected by the naive Bayesian classification. Finally, WKNN is used to estimate the position.

## 3. The Proposed Positioning Algorithm

The state spaces, naive Bayesian, and Position Estimation Algorithm will be introduced in this section.

### 3.1. Description and Solution of RPCA

In any state space, the offline fingerprint of the *i*-th RP can be expressed by (1). Assuming each AP is sampled *Q* times, all collected data from the *M*-th AP at the *i*-th RP could be expressed as follows:

(3) $$rss_{i,M}={\left({rssi}_{1,M},{rssi}_{2,M},\cdots ,{rssi}_{Q,M}\right)}^{T},$$

where ${rssi}_{Q,M}$ represents the *S*-th sample for the *M*-th AP at the *i*-th RP. Generally, RSSI should exceed −110 dBm because, due to the receiver sensitivity conditions, signals below this threshold cannot be detected [30]. However, actual measurements indicate that signals below −95 dBm are difficult to detect, which means −95 dBm could replace the missing data. Thus, the data collected from all APs at the i-th RP could be written as follows:

(4) $${\tilde{\phi }}_{i}={\left(\begin{array}{c} rssi_{1,1},rssi_{1,2},\cdots ,rssi_{1,M}\\ rssi_{2,1},rssi_{2,2},\cdots ,rssi_{2,M}\\ \vdots \\ rssi_{Q,1},rssi_{Q,2},\cdots ,rssi_{Q,M} \end{array}\right)}_{Q\times M}.$$

Generally, fingerprint information is measured by the average level of the real data, specifically by calculating the mean of the sampled data, as shown below:

(5) $${\bar{\phi }}_{i}=\left({\bar{rss}}_{i,1},{\bar{rss}}_{i,2},\cdots ,{\bar{rss}}_{i,M}\right),i\in 1,2,\cdots ,N.$$

To minimize errors caused by noise, this paper uses RPCA to process the collected data. The detailed process is described below. Since SVD matrix decomposition of RPCA requires a square matrix, all sampled data must be reconstructed. The convex optimization model of RPCA could be seen from (7), where λ is a balance factor to control the weight between two output matrices. For n×m input data, the best choice for λ is $m^{-1/2}$ or $n^{-1/2}$. A larger discrepancy between *m* and *n* worsens RPCA results, making a square matrix ideal for balance. Assuming the total number of samples from the AP is *Q*, where $Q=q^{2},q\in N^{+}$, *Q* should be such that its square root is an integer to allow the sampled data to be rearranged into a square matrix. The data in (3) can be rearranged as follows:

(6) $$R={\left(\begin{array}{c} rssi_{1},rssi_{q+1},\cdots ,rssi_{(q-1)\times q+1}\\ rssi_{2},rssi_{q+2},\cdots ,rssi_{(q-1)\times q+2}\\ \vdots \\ rssi_{s},rssi_{2\times q},\cdots ,rssi_{q\times q} \end{array}\right)}_{q\times q}.$$

The concept of RPCA [26] is to decompose the initial matrix R into the sum of two matrices: $\hat{R}$ and E. Here, $\hat{R}$ is a low-rank matrix, and outlier noise is the other matrix E. Thus, $\hat{R}$ contains the clean data needed after noise reduction. The collected data from two APs are shown in Figure 2. In the figure, it is clearly visible that there are numerous outliers in addition to the main data trajectory, which aligns well with the RPCA modeling concept.

Next, by solving the optimization problem constructed by the RPCA model, a low-rank matrix $\hat{R}$ and a noise matrix E can be obtained. The low-rank matrix $\hat{R}$ represents the denoised data. This optimization model can be expressed in the following form:

(7) $$\begin{array}{c} \min_{\hat{R},E}\;\;rank\left(\hat{R}\right)+\lambda {∥E∥}_{0}\\ \mathrm{subject}\;\mathrm{to}\;R=\hat{R}+E \end{array}.$$

In Equation (7), $rank(\hat{R})$ is the rank of matrix $\hat{R}$ and ${∥\cdot ∥}_{0}$ is the number of nonzero elements in the matrix. Additionally, the balance between $\hat{R}$ and E is adjusted through λ>0. Since both the matrix rank and the $\ell_{0}$-norm are non-convex, the optimization problem in (7) is NP-hard. To address this, the elements in the equation need to be scaled. The nuclear norm and $\ell_{1}$-norm serve as the convex hulls for the rank and $\ell_{0}$-norm, respectively [31,32]. Therefore, the problem can be reformulated as the following convex optimization problem:

(8) $$\begin{array}{c} \min_{\hat{R},E}\;\;{∥\hat{R}∥}_{*}+\lambda {∥E∥}_{1}\\ \mathrm{subject}\;\mathrm{to}\;R=\hat{R}+E \end{array}.$$

Since it has become a convex optimization problem, a minimum value can be obtained. This can be solved using an Inexact Augmented Lagrange Multiplier (IALM) method [29].

The form of the augmented Lagrangian function can be expressed as follows:

(9) $$\begin{array}{cc} L(\hat{R},E,Y,\mu ) & ={∥\hat{R}∥}_{*}+\lambda {∥E∥}_{1}+\langle Y,R-\hat{R}-E\rangle +\frac{\mu }{2}{∥R-\hat{R}-E∥}_{F}^{2}. \end{array}$$

where the Y is a Lagrange multiplier and μ is a positive scalar. The IALM method alternates iterations between the matrices $\hat{R}$ and E until convergence. The update formula for $\hat{R}$, E, and Y used in the algorithm iteration can be expressed as follows:

(10) $$\begin{array}{cc} {\hat{R}}_{k+1} & =\underset{\hat{R}}{\arg\;\min}\;L\left(\hat{R},E_{k+1},Y_{k},\mu_{k}\right)\\ \; & =\underset{\hat{R}}{\arg\;\min}\;{∥\hat{R}∥}_{*}+\frac{\mu_{k}}{2}{∥\hat{R}-\left(R-E_{k+1}+\frac{Y_{k}}{\mu_{k}}\right)∥}_{F}^{2}, \end{array}$$

(11) $$\begin{array}{cc} E_{k+1} & =\underset{E}{\arg\;\min}\;L\left({\hat{R}}_{k+1},E,Y_{k},\mu_{k}\right)\\ \; & =\underset{E}{\arg\;\min}\;\lambda {∥E∥}_{1}+\frac{\mu_{k}}{2}{∥E-(R-{\hat{R}}_{k+1}+\frac{Y_{k}}{\mu_{k}})∥}_{F}^{2}, \end{array}$$

(12) $$Y_{k+1}=Y_{k}+\mu_{k}\left(R-{\hat{R}}_{k+1}-E_{k+1}\right).$$

The detailed algorithmic process for solving RPCA using the IALM method is shown in Algorithm 1. The initialization parameter $Y_{0}$ of the algorithm is

(13) $$Y_{0}=R/J\left(R\right),$$

where the calculation method of J(R) can refer to the following Equation (14), and R comes from Equation (6).

(14) $$J\left(R\right)=\max\left({∥R∥}_{2},\lambda^{-1}{∥R∥}_{\infty }\right).$$

The low-rank matrix $\hat{R}$ can be shown as

(15) $$\hat{R}={\left(\begin{array}{c} r\hat{ss}i_{1},r\hat{ss}i_{q+1},\cdots ,r\hat{ss}i_{(q-1)\times q+1}\\ r\hat{ss}i_{2},r\hat{ss}i_{q+2},\cdots ,r\hat{ss}i_{(q-1)\times q+2}\\ \vdots \\ r\hat{ss}i_{q},r\hat{ss}i_{2\times q},\cdots ,r\hat{ss}i_{q\times q} \end{array}\right)}_{q\times q}.$$

After obtaining $\hat{R}$, it can be unfolded to recover the original sampled data format, as shown below:

(16) $${\hat{r}}_{i,M}={\left({r\hat{ss}i}_{1,M},{r\hat{ss}i}_{2,M},\cdots ,{r\hat{ss}i}_{Q,M}\right)}^{T},$$

Following noise reduction by RPCA, the fingerprint of an AP in the *j*-th state space for this RP could be expressed (16), obtained by the mean of the processed data. This processing method is applied to all RSSI sampling data. As a result, the offline database, processed through RPCA, could be constructed as

(17) $${\hat{\Phi }}_{j}=\left(\begin{array}{c} {\hat{\phi }}_{1}j\\ {\hat{\phi }}_{2}j\\ \vdots \\ {\hat{\phi }}_{N}j \end{array}\right)={\left(\begin{array}{c} {r\hat{s}s}_{1,1}^{j},{r\hat{s}s}_{1,2}^{j},\cdots ,{r\hat{s}s}_{1,M}^{j}\\ {r\hat{s}s}_{2,1}^{j},{r\hat{s}s}_{2,2}^{j},\cdots ,{r\hat{s}s}_{2,M}^{j}\\ \vdots \\ {r\hat{s}s}_{N,1}^{j},{r\hat{s}s}_{N,2}^{j},\cdots ,{r\hat{s}s}_{N,M}^{j} \end{array}\right)}_{N\times M}.$$

**Algorithm 1** RPCA Solution by IALM
**Input:** Original matrix $R\in R^{s\times s}$, $\lambda =s^{-1/2}$
  1:$Y_{0}=R/J\left(R\right);E_{0}=0;\mu_{0}>0;\rho >1;k=0.$  2:**while** not converged **do**  3:   $\left(U,S,V\right)=svd\left(R-E_{k}+\mu_{k}^{-1}Y_{k}\right);$  4:   ${\hat{R}}_{k+1}=US_{\mu_{k}^{-1}}\left[S\right]V^{T};$  5:   $E_{k+1}=S_{\lambda \mu_{k}^{-1}}\left[R-{\hat{R}}_{k+1}+\mu_{k}^{-1}Y_{k}\right];$  6:   $Y_{k+1}=Y_{k}+\mu_{k}\left(R-{\hat{R}}_{k+1}-E_{k+1}\right);$  7:   $\mathrm{Update}\;\mu_{k}\;\mathrm{to}\;\mu_{k+1};$  8:   k←k+1;  9:**end while****Output:** ${\hat{R}}_{k},E_{k}$


### 3.2. State Spaces

Assuming that there are *H* doors that can change the spatial connectivity in the experimental space, the total number of space states *S* is obtained by enumerating all possible door opening and closing situations. It can be expressed as

(18) $$S=C_{H}^{0}+C_{H}^{1}+\cdots +C_{H}^{h}+\cdots +C_{H}^{H},H\in N^{+}.$$

where $C_{H}^{h}$ means that any *h* doors among the *H* doors are open. According to the binomial theorem,

(19) $${\left(x+y\right)}^{n}=C_{n}^{0}x^{n}y^{0}+C_{n}^{1}x^{n-1}y^{1}+\cdots +C_{n}^{n}x^{0}y^{n}.$$

When x=1, y=1; then, letting n=H, (19) can be simplified as

(20) $$2^{H}=C_{H}^{0}+C_{H}^{1}+\cdots +C_{H}^{H}=S.$$

**Figure 3:**
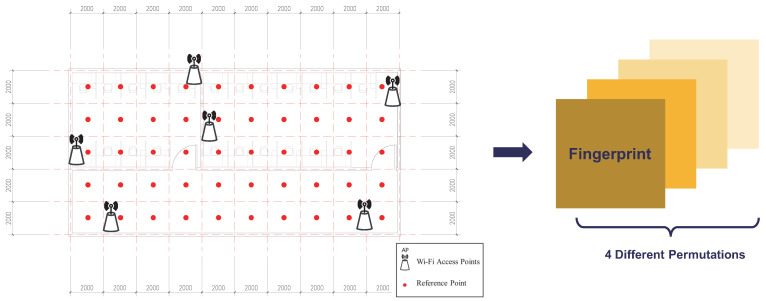
Based on all the different door opening and closing situations, identify all the possible permutations that affect the AP states and use this information to construct a multi-fingerprints database that includes all possible scenarios.

For example, there is an experimental space shown in Figure 3, with 50 RPs, 10 APs, and 2 doors. If ‘0’ represents a closed door and ‘1’ represents an open door, it can be easily seen that there are four spatial states, which are {00,01,10,11}, in the environment. All the fingerprints of different AP states are placed into their corresponding state spaces to construct a multi-fingerprints database according to (2): $\Sigma =(\Phi_{1},\Phi_{2},\cdots ,\Phi_{S})$.

It is worth noting that the values of AP in different state spaces are not always changed, which depends on the location of the RP that receives the signal strength of the AP. If the RP is in the same room as the AP, regardless of how the state space changes, the RP receiving the RSSI of this AP remains unchanged. This is because the original transmission path has not been affected by the change in the spatial state and is still a line-of-sight transmission.

### 3.3. Choose State Space by Naive Bayesian

Naive Bayesian is a classification method under the probability framework [33]. The core idea is to obtain the minimum conditional risk of each sample as the class label, which is also known as the posterior probability. The posterior probability of the online fingerprint being in each state space is calculated using Bayes’ theorem; then, the state space with the maximum posterior probability is selected.

Assume that the online fingerprint is $\Psi =(\psi_{1},\psi_{2},\cdots ,\psi_{M})$. According to Bayes’ theorem, the posterior probability that Ψ belongs to *j*-th state space $\Phi_{j}$ is

(21) $$P\left(\Phi_{j}\left|\Psi \right.\right)=\frac{P\left(\Psi \left|\Phi_{j}\right.\right)P\left(\Phi_{j}\right)}{P(\Psi )},$$

where $P(\Phi_{j})$ is the prior probability of state space $\Phi_{j}$, $P(\Psi \left|\Phi_{j}\right.)$ is the probability of online fingerprint Ψ appearing under the condition of given state space $\Phi_{j}$, P(Ψ) is the marginal probability that online fingerprint Ψ occurs, and j∈{1,2,⋯,S}. Since P(Ψ) in (21) does not vary with changes in the state space and all state spaces have the same size, i.e., the prior probability $P(\Phi_{j})$ is the same, it is only necessary to obtain the $P(\Psi \left|\Phi_{j}\right.)$ part in this paper.

(22) $$P\left(\Psi \left|\Phi_{j}\right.\right)=P\left(\psi_{1},\psi_{2},\cdots ,\psi_{M}\left|\Phi_{j}\right.\right),$$

where $\psi_{M}$ is the online RSSI from the *M*-th AP. According to the assumption of naive Bayes, each AP is independent. Therefore, $P(\Psi \left|\Phi_{j}\right.)$ can be represented as the product of conditional probabilities of each AP, i.e.,

(23) $$\begin{array}{cc} P(\Psi \left|\Phi_{j}\right.) & =\prod_{i=1}^{M}P(\psi_{i}\left|\Phi_{j}\right.)\\ \; & =\prod_{i=1}^{M}\frac{1}{\sqrt{2\pi }\sigma_{ij}}\exp\left(-\frac{{\left(\psi_{i}-\mu_{ij}\right)}^{2}}{2\sigma_{ij}^{2}}\right), \end{array}$$

where $\sigma_{ij}$ and $\mu_{ij}$ are the standard deviation and mean of all values of the *i*-th AP in the *j*-th state space in the offline database, respectively. The state space to which the online fingerprint belongs is selected with the class with the largest posterior probability $\zeta =\arg\max_{j\in \{1,2,\cdots ,S\}}P\left(\Psi \left|\Phi_{j}\right.\right)$.

### 3.4. Position Estimation

Once the state space corresponding to the online fingerprint is selected through the naive Bayes classifier, traditional fingerprint localization algorithms such as KNN and WKNN can be used for position estimation. The similarity between the online fingerprint and the fingerprints of all RPs in the selected state space is measured by calculating the Euclidean distance. The resulting similarity vector *D* is as follows:

(24) $$D=(d_{1},d_{2},\cdots ,d_{N}),$$

where $d_{N}$ denote the Euclidean distance between the offline and online fingerprint of the *N*-th RP. According to the offline fingerprint in (2), $d_{N}$ could be expressed as

(25) $$d_{N}=\sqrt{\sum_{i=1}^{M}{\left(\psi_{i}-rss_{Ni}^{\zeta }\right)}^{2}},$$

where $rss_{Ni}^{\zeta }$ denote the offline fingerprint from the *i*-th AP on the *N*-th RP in the state space with the highest probability. The position estimate can be obtained by WKNN:

(26) $$\hat{L}\left(x,y\right)=\sum_{i=1}^{K}l_{i}\left(x_{i},y_{i}\right)\times w_{i},$$

where $w_{i}$ is determined by

(27) wi=1di1di∑j=1K1dj∑j=1K1dj.

## 4. Simulation and Experimental Analysis

The two laboratories and their connecting corridor in Building 2 of Nanjing University of Information Science and Technology were utilized as the test space. The test space layout is depicted on the left side of Figure 3. The detailed configuration is presented in Table 1.

**Figure 4:**
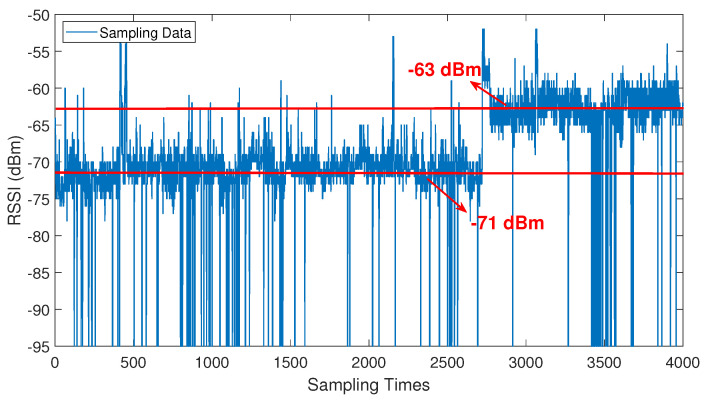
A total of 4000 samples were collected at a reference point for a single AP.

Figure 4 presents the long-term sampling data for a specific AP at a reference point. From the two red lines in the graph, it can be observed that the reference point experienced two different spatial states during the 4000 sampling process, with average RSSI values of −63 dBm and −71 dBm, respectively.

**Figure 5:**
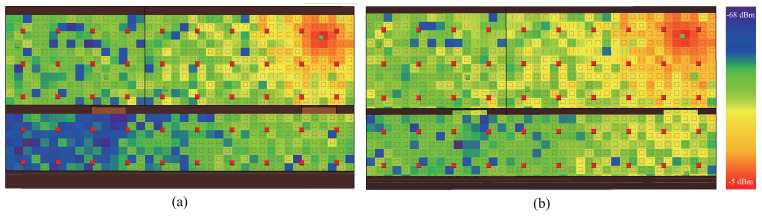
Under the spatial layout of the experimental area, a commercial software, “Wireless Insite 3.4.4”, was utilized to simulate and compare the signal propagation of an AP in different spatial configurations. (**a**) Corresponds to the scenario when the doors of both laboratories are closed. (**b**) Corresponds to the scenario when the doors of both laboratories are open. The red points represent reference points while the green one is the AP.

This variation is attributed to the opening and closing of the laboratory doors, which directly influenced the transmission quality of the Wi-Fi signal. This finding confirms that changes in the environmental spatial state can significantly impact the signal of the AP. Moreover, Wireless Insite was employed to simulate the radiation pattern of the AP signal in a spatial layout identical to the experimental environment, further validating the aforementioned findings. In comparison to Figure 5a, the simulation results from Figure 5b showed significantly improved overall signal quality of the AP throughout the entire experimental area, especially in the lower-left corner, which represents the farther end from the AP. When both laboratory doors were closed, the signal quality in the lower-left corner was notably poor, with the majority of locations having an RSSI below −65 dBm. However, when both laboratory doors were opened, it can be observed that the signal quality in the lower-left corner improved significantly, with most locations reaching a level of approximately −40 dBm. Additionally, there was a corresponding increase in signal strength at other locations. This result further confirms the significant variation in AP signal strength under different spatial environmental states.

**Figure 6:**
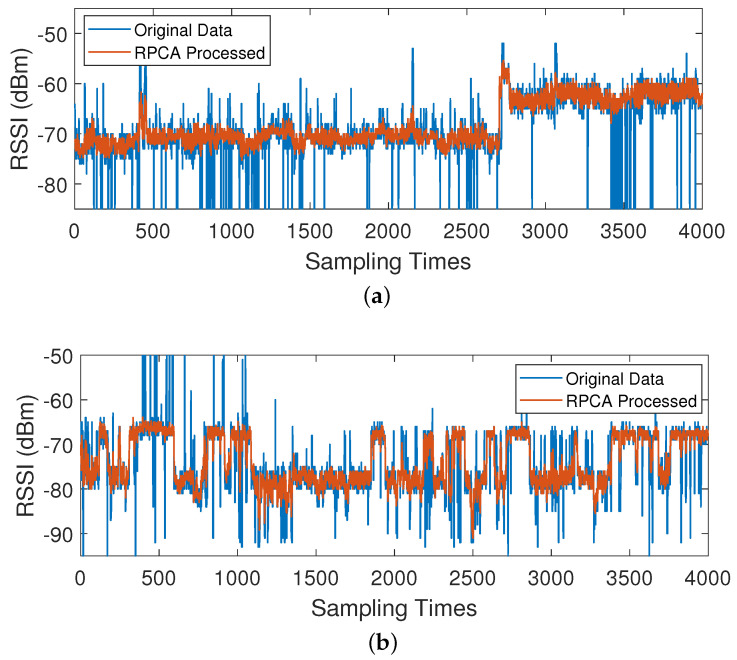
(**a**,**b**) Represent a comparison of the results before and after RPCA denoising for 4000 sampling data from two APs.

Figure 6 shows a comparison of the results after RPCA denoising of the sampled data. The blue part represents the original sampled data while the orange part represents the data after RPCA denoising. The results show that RPCA significantly reduces noise in the actual measurement data, filtering out a substantial number of outliers.

**Figure 7:**
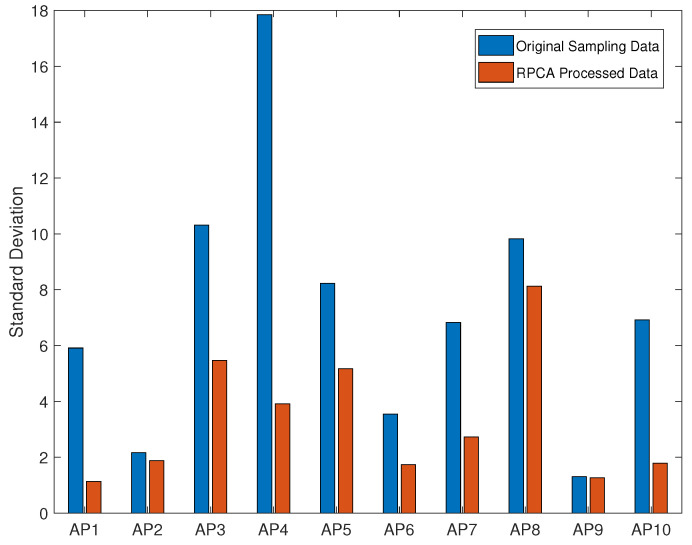
The standard deviation of data for an RP.

Figure 7 compares the standard deviations of two datasets. The comparison reveals that the standard deviation of the data processed with RPCA is lower than the data without processing. This supports the validity of the earlier conclusions and demonstrates the effectiveness of RPCA in handling outlier data. Thus, using RPCA for noise reduction is practical in engineering applications.

**Figure 8:**
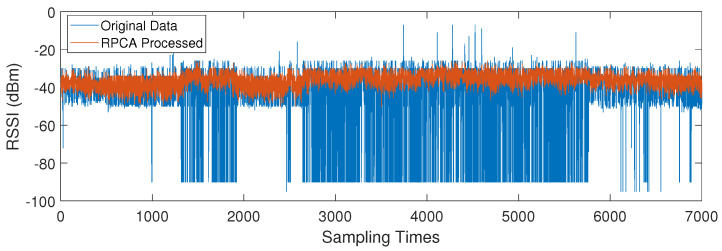
Comparison of AP4 sampling data before and after RPCA processing.

Table 2 provides the fingerprint data of all 10 APs for a reference point; the unit of all data is dBm. From the table, it can be observed that the fingerprints processed with RPCA are more accurate than the normal fingerprints. Combining Figure 7 and Table 2, it can be observed that the more stable the AP received signal, the smaller the difference between RPCA-processed fingerprints and normal fingerprints. For example, AP9 has a robust RSSI signal with almost no outlier noise, which could obtain excellent fingerprints even without RPCA processing. Conversely, for AP4, the excessive signal fluctuations generate a large amount of outlier noise, and RPCA performs well on the data from this AP. Through the RPCA, the standard deviation of AP4 data decreased from 17.85 to 3.91, a reduction of 78.07%. Its fingerprint improved from −42.55 dBm to −36.11 dBm, an increase of 15.13%. The details of the AP4 data are shown in the Figure 8. From the orange results, it can be seen that a lot of outlier noise is filtered out, enhancing the robustness of the data.

**Figure 9:**
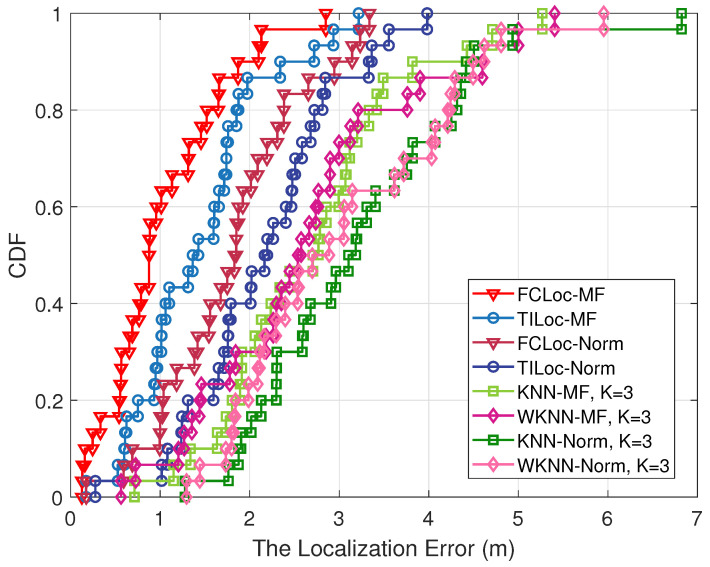
CDF of the localization errors.

Four different fingerprint-based localization methods were selected to evaluate the positioning performance on both the multi-fingerprints database and the traditional single-fingerprint database. The selected algorithms include the newer FCLoc [16] and TILoc [28] algorithms as well as the classic KNN and WKNN algorithms. The cumulative distribution function (CDF) of the localization errors for all algorithms are presented in Figure 9. Among them, “MF” represents the mode using the multi-fingerprints database, while “Norm” denotes the traditional mean-based fingerprint database.

Table 3 provides detailed statistics, with all values expressed in meters (m). Combining Table 3 with Figure 9, it can be concluded that 80% of the FCLoc-MF positioning errors are within 1.52 m and the average error is 1.02 m. Meanwhile, 80% of the FCLoc-Norm localization errors are within 2.38 m, and the mean error is 1.68 m. The TILoc, KNN, and WKNN algorithms utilizing the “MF” multi-fingerprints database achieved 80% localization errors within 1.85 m, 3.33 m, and 3.21 m, respectively; their mean localization errors were 1.44 m, 2.68 m, and 2.63 m, respectively. In contrast, using the traditional “Norm” fingerprint database, the 80% localization errors were within 2.72 m, 4.32 m, and 4.21 m, with mean errors of 2.16 m, 3.74 m, and 3.46 m, respectively. The FCLoc algorithm showed an improvement of approximately 39% in localization performance when using the multi-fingerprints approach. TILoc, KNN, and WKNN demonstrated performance improvements of approximately 33%, 28%, and 24%, respectively.

## 5. Conclusions

Currently, research on indoor localization based on fingerprints predominantly focuses on various solutions based on single fingerprints. However, based on practical research experience, it has been observed that the variation in spatial environmental conditions significantly affects fingerprints, making a single fingerprint inadequate for representing different spatial states. To alleviate this issue and improve system performance by enhancing fingerprint matching accuracy, this paper proposes the core concept of constructing a multi-fingerprints database based on different spatial environmental states. By analyzing potential spatial structural changes in the entire experimental space, different fingerprints are mapped to distinct state spaces to establish the multi-fingerprints database. During the online phase, a naive Bayesian approach is employed to analyze the state space in which real-time fingerprints reside, enabling the selection of the corresponding fingerprint database for matching. Through testing in the selected experimental area, this paper demonstrates the performance of different localization algorithms on both the multi-fingerprints database and traditional fingerprint database. The experimental results show significant improvements in the performance of the tested algorithms using the proposed multi-fingerprints selection strategy. Meanwhile, this paper uses RPCA denoising to obtain a more accurate fingerprint database. Experimental results demonstrate that RPCA effectively addresses the impact of sparse noise. In fact, the multi-fingerprints localization strategy has significant research potential. The multi-fingerprints database construction scheme based on environmental state space proposed in this paper still has obvious shortcomings. For example, if the experimental environment is expanded, the magnitude of the state space will grow exponentially. Additionally, this paper only proposes a solution based on naive Bayesian analysis though, clearly, there are more options available, such as probabilistic linear discriminant analysis and machine learning-based approaches. Therefore, we propose the following considerations for future work:

- Find simpler and more effective methods for constructing and online multi-fingerprint matching to address broader and more general application scenarios.
- Consider more complex factors in multi-fingerprint construction, not just limited to environmental state space but also including factors like personnel movement and object relocation.
- Explore whether other data sources exhibit characteristics of multiple states similar to RSSI, and build a richer, more comprehensive database through the integration of different data sources.

## Figures and Tables

**Table 1:** Test Configurations.

Object	Setting
Area size	20 m × 10 m
Grid size	2 m × 2 m
Test Software	WirelessMon V4.0
Sampling frequency	0.125 Hz
Number of APs	10
Number of RPs	50
Height of RPs	1.5 m
Test equipment	HUAWEI MateBook 14
Offline sampling duration	Over 8 h per RP
Online sampling duration	90 s per TP

**Table 2:** All APs’ fingerprints for a reference point.

Fingerprint Type	AP1	AP2	AP3	AP4	AP5	AP6	AP7	AP8	AP9	AP10
Normal_Fingerprint	−58.30	−69.15	−78.14	−42.55	−73.83	−84.48	−70.79	−86.80	−33.74	−48.10
RPCA_Fingerprint	−57.54	−68.99	−77.30	−36.11	−73.23	−83.66	−70.32	−86.60	−33.68	−47.33

**Table 3:** Real test results of localization errors.

Algorithm	Min	Max	Mean
FCLoc-MF	0.13	2.85	1.02
FCLoc-Norm	0.17	3.34	1.68
TILoc-MF	0.16	3.22	1.44
TILoc-Norm	0.28	3.98	2.16
KNN-MF, K = 3	0.71	5.27	2.68
KNN-Norm, K = 3	1.29	6.42	3.74
WKNN-MF, K = 3	0.56	5.41	2.63
WKNN-Norm, K = 3	1.31	6.24	3.46

## Data Availability

Data available upon request from the authors if the paper is accepted.
